# The Effects of Circadian Rhythm on Lead-Induced Toxicity in the DBC1.2 Olfactory Dark Basal Cell Line

**DOI:** 10.3390/cells14020081

**Published:** 2025-01-09

**Authors:** Teru Kamogashira, Shu Kikuta, Tatsuya Yamasoba

**Affiliations:** 1 Department of Otolaryngology and Head and Neck Surgery, Faculty of Medicine, University of Tokyo, Tokyo 113-8655, Japan; 2 Department of Otolaryngology and Head and Neck Surgery, Faculty of Medicine, Nihon University, Tokyo 173-8610, Japan; 3 Department of Otolaryngology, Tokyo Teishin Hospital, Tokyo 102-0071, Japan

**Keywords:** lead, Pb, olfactory epithelium, circadian rhythm, mitochondrial function

## Abstract

Background/Objectives: This study evaluated changes in circadian clock genes and mitochondrial function in a lead (Pb)-induced toxicity model of an olfactory epithelial cell line. Methods: The DBC1.2 olfactory dark basal cell line was used. Dexamethasone shock was used to reset the circadian clock 24 h (Group 1) and 36 h (Group 2) after seeding. Then, 60 h after seeding, the cells were treated with or without Pb (II) nitrate in HEPES buffer for 1 h. Mitochondrial function and cell viability were evaluated 84 h after seeding. Results: Mitochondrial function under Pb exposure was significantly impaired in Group 1 compared with Group 2. Cell numbers and viability did not significantly differ between groups. The mitochondrial membrane potential was significantly higher in Group 1 than Group 2, both without and with Pb exposure. Conclusions: The circadian rhythm can alter the sensitivity to Pb-induced toxicity and mitochondrial damage in olfactory cells.

## 1. Introduction

The circadian rhythm is controlled by an internal timing system that is regulated at the transcriptional level, creating a gene network that oscillates in a 24 h cycle, and the core molecular components of the mammalian circadian clock include genes such as Clock, Brain and muscle Arnt (Aryl hydrocarbon receptor nuclear translocator)-like protein-1 (Bmal1), Period (Per) (Per1, Per2, and Per3), and Cryptochrome (Cry) (Cry1 and Cry2) [1]. These genes interact in a tightly regulated feedback loop, where Clock and Bmal1 activate the transcription of Per and Cry, which in turn inhibit their own expression, thus creating a self-sustaining cycle. These genes follow a roughly daily cycle, synchronizing with environmental cues such as light and temperature to regulate an organism’s physiological and behavioral rhythms. This network includes clock genes that control physiological functions and behavioral rhythms. Recent research has revealed that these genes not only govern daily rhythms in sleep–wake cycles but are also deeply involved in cell proliferation and mitochondrial function, and abnormalities in these genes are related to sleep disorders, cancer, Alzheimer’s disease, and aging [2,3].

Recent studies have highlighted the significance of olfactory dysfunction in dementia and neurological disorders including Alzheimer’s disease [4,5,6], emphasizing the importance of olfactory function in the aging process. The olfactory epithelium (OE) is exposed to the external environment and is easily damaged by harmful factors such as viruses [7]. Basal cells, which are involved in the regeneration of the OE, express circadian clock genes, and thus circadian rhythms may change the susceptibility of basal cells to injury and cell function [8,9]. However, these changes have not yet been experimentally proven.

We previously established a lead (Pb)-induced toxicity model in the Dark basal cells 1.2 (DBC1.2) olfactory dark basal cell line and found that endoplasmic reticulum (ER) stress and cell damage cause necroptosis [10]. DBC1.2 is a cell line derived from DBCs, which are also called horizontal basal cells (HBCs), derived from a primary culture of the OE of newborn mice from embryonic day 14.5 [11]. Pb induces cellular damage by promoting reactive oxygen species (ROS) production with DNA damage [12], mitochondrial dysfunction [13], and endoplasmic reticulum (ER) stress [14]. These effects disrupt cellular homeostasis and contribute to toxicity. ER stress occurs when the folding capacity of the ER is overwhelmed, leading to the accumulation of misfolded or unfolded proteins. This triggers the unfolded protein response (UPR), which restores homeostasis or initiates apoptosis if the stress is unresolved. Circadian clock genes, such as Clock and Bmal1, regulate the expression of UPR-related proteins, including chaperones like binding immunoglobulin protein (BiP)/glucose regulating protein 78 (Grp78) and components of the protein kinase R (Pkr)-like endoplasmic reticulum kinase (Perk)-eukaryotic initiation factor 2α (eIF2α) signaling pathway [15], and thus circadian rhythm fluctuations may affect basal cell function via ER stress. Necroptosis, a form of programmed necrotic cell death mediated by receptor-interacting protein kinases (Ripk1 and Ripk3) and mixed lineage kinase domain-like protein (Mlkl) [16], is also influenced by circadian clock genes [17]. Evidence suggests that the rhythmic expression of necroptosis-related genes is coordinated by the circadian clock, affecting cellular susceptibility to necroptotic triggers. Mitochondria are closely involved in both ER stress [18] and necroptosis [19], serving as a central hub for their interrelated signaling pathways.

In this study, we investigated mitochondrial function and circadian clock gene changes in the DBC1.2 Pb-induced toxicity model and the effects of circadian rhythm on basal cell damage.

## 2. Materials and Methods

### 2.1. Cell Cultures

DBC1.2 cells were purchased from the Japanese Collection of Research Bioresources (JCRB) Cell Bank, National Institutes of Biomedical Innovation, Health and Nutrition. The cells were cultured in Dulbecco’s Modified Eagle Medium/nutrient mixture F-12 (DMEM/F-12) (08460-95; Nacalai Tesque, Kyoto, Japan, 042-30555; FUJIFILM Wako Pure Chemical Corporation, Osaka, Japan) containing 10% fetal bovine serum (FBS) (26140079; Life Technologies, Inc., Waltham, MA, USA) and penicillin (100 units/mL) with streptomycin (100 μg/mL; 168-23191; FUJIFILM Wako Pure Chemical Corporation, Osaka, Japan) [10]. Cells were incubated at 37 °C with 5% CO_2_.

### 2.2. Pb Exposure

Pb (II) nitrate (129-03222; FUJIFILM Wako Pure Chemical Corporation, Osaka, Japan) was dissolved in HEPES buffer [20]. The composition of HEPES buffer was modified based on HEPES–Ringer (Krebs–Ringer solution with HEPES): 140 mM NaCl, 5 mM KCl, 2 mM CaCl_2_, 1 mM MgCl_2_, 5 mM HEPES (GB10 HEPES; Dojindo, Kumamoto, Japan), and 10 mM D-Glucose.

### 2.3. Cell Viability Assay and Population Analysis

Cell viability assays were performed using WST-8 (CK04 Cell Counting Kit-8; Dojindo, Kumamoto, Japan, 07553-44; Cell Count Reagent SF, Nacalai Tesque, Kyoto, Japan). Cells were incubated with WST-8 for 90 min according to the manufacturer’s instructions, and their absorbance was measured at 450 nm and 600 nm (for noise reduction) using the ChroMate 4300 plate reader (Awareness Technologies, Westport, CT, USA). Cells in the plate (4000 cells/well at time of passage) were washed once with phosphate-buffered saline (PBS), fixed with 4% paraformaldehyde (PFA) (37152-64; 10%-Formaldehyde Neutral Buffer Solution pH6.9–7.1, Nacalai Tesque, Kyoto, Japan) for 1 min, stained with Hoechst 33,342 (final concentration: 5 µg/mL; H1399; Thermo Fisher Scientific Inc., Waltham, MA, USA) for 30 min, and then washed again with PBS. Cells were observed with a fluorescence microscope (BZ-X710; KEYENCE, Osaka, Japan) using a DAPI filter cube and counted automatically using ImageJ 1.54m [21,22] with Fiji 2.14.0 [23] (Appendix A).

### 2.4. Quantitative PCR Analysis

Total RNA was isolated with NucleoSpin RNA/Protein (740933.50; Macherey-Nagel, Düren, Germany) according to the manufacturer’s instructions. The concentration of extracted mRNA was measured using a NanoDrop Lite (Thermo Fisher Scientific Inc., Waltham, MA, USA), and complementary DNA (cDNA) was generated using ReverTraAce qPCR RT Master Mix with gDNA Remover (FSQ-301; Toyobo, Osaka, Japan) according to the manufacturer’s instructions. The quality of mRNA was assessed by visualization of the 28S and 18S ribosomal RNA bands using DynaMarker, RNA High for Easy Electrophoresis (DM170; BioDynamics Laboratory Inc., Tokyo, Japan). The primers and probes [24] (Appendix B, final concentration: forward: 500 nM, reverse: 500 nM, probe: 250 nM) were purchased from FASMAC (Kanagawa, Japan). The forward and reverse primers were mixed with cDNA (at a final concentration of 400 ng/20 µL/well), and quantitative PCR was performed using THUNDERBIRD Probe qPCR Mix (QPS-101; Toyobo, Osaka, Japan). The following experimental run protocol was used: denaturation and activation program at 95 °C for 60 s and amplification and quantification program repeated 60 times (95 °C for 15 s and 60 °C for 45 s). Data collection was performed using the QuantStudio 7 Flex Detection System (Thermo Fisher Scientific Inc., Waltham, MA, USA). The 2^−ΔΔCt^ method was applied to analyze the relative changes in gene expression. The mRNA expression level of each gene was normalized using GAPDH as an internal control.

### 2.5. Extracellular Flux Analysis

Cells were seeded into the 24-well microplates of an XF24 Extracellular Flux Analyzer (100777-004; Seahorse Bioscience, Billerica, MA, USA) (8000 cells/well in the long-term observation and 70,000 cells/well in the evaluation under lead (Pb) exposure) with 4 blank background wells according to the Seahorse XF24 User’s Manual. As shown in Figure 2, following vehicle injection or dexamethasone shock, the cells were rinsed twice well, including wall surfaces, with 1 mL Dulbecco’s PBS (D-PBS) (-) (14249-24, 14249-95; Nacalai Tesque, Kyoto, Japan, 045-29795, 049-29793; FUJIFILM Wako Pure Chemical Corporation, Osaka, Japan) and once with 500 µL XF assay medium (DMEM without NaHCO_3_, 103335-100; Seahorse Bioscience, Billerica, MA, USA). They were then re-suspended in 1200 µL XF assay medium, supplemented with 25 mM D-glucose (Otsuka Seiyaku, Tokushima, Japan), 1 mM sodium pyruvate (06977-34; Nacalai Tesque, Kyoto, Japan), and 2 mM L-Glutamine (103579-100; Seahorse Bioscience, Billerica, MA, USA). After the cells were equilibrated overnight at 37 °C in a non-CO_2_ incubator prior to the assay, the oxygen consumption rate (OCR) was measured using the XF24 Extracellular Flux Analyzer (Seahorse Bioscience, Billerica, MA, USA). To eliminate the effect of the medium replacement, the cells were incubated overnight in a non-CO_2_ incubator after the medium was replaced with the assay medium. As shown in Figures 4 and 5, the cells were rinsed as above and re-suspended in 675 µL XF assay medium supplemented as above, equilibrated for 1 h as above, and were then submitted to the assay.

The cells were subjected to the following additions (in sequence): (1) basal levels were measured with no additives; (2) oligomycin (at a final concentration of 0.6 µM) (4110/5, 5 mg; Tocris Bioscience, Bristol, UK) was added to inhibit ATP synthase and oxidative phosphorylation; (3) carbonyl cyanide-4-(trifluoromethoxy) phenylhydrazone (FCCP) (at a final concentration of 1 µM) (0453/10, 10 mg; Tocris Bioscience, Bristol, UK) was added to uncouple the proton gradient and induce maximal respiration; (4) antimycin A (at a final concentration of 0.16 µM) (A8674-25MG; Sigma Aldrich, Inc., St. Louis, MO, USA) was added to inhibit complex III; and (5) rotenone (at a final concentration of 0.125 µM) (30227-04, 5 g; Nacalai Tesque, Inc., Kyoto, Japan) was added to inhibit complex I. Four separate measurements of the OCR and extracellular acidification rate (ECAR) were taken after the addition of each inhibitor.

Optimization of cell density and working concentration titers for each inhibitor were completed prior to the analysis according to the Seahorse XF24 User’s Manual. The OCR and ECAR were automatically calculated, recorded, and plotted by Seahorse XF24 software (version 1.8.1.01). The number of cells in each well was analyzed after the flux assay using the protocol above to normalize OCR values.

### 2.6. Mitochondrial Membrane Potential (MMP) Assay

MMP was measured using the mitochondrial membrane fluorescent dye 5,5′,6,6′-tetrachloro-1,1′,3,3′-tetraethyl benzimidazolyl carbocyanine iodide (JC-1) (#70014; Biotium Inc., Fremont, CA, USA). JC-1 is taken up by mitochondria and forms either JC-1 dimer aggregates that emit red fluorescence at high membrane potentials or JC-1 monomers that emit green fluorescence at low membrane potentials. Cells were seeded into 24-well microplates (µ-Plate 24 Well ibiTreat, ib82426; ibidi GmbH, Gräfelfing, Germany) and exposed to Pb. Cells were incubated with medium with JC-1 (200 nM) for 30 min, and then the medium was replaced with normal medium before the MMP ratio measurements. The fluorescence emission ratio was analyzed with an infinite M200 PRO monochromator plate reader (Tecan Group Ltd., Männedorf, Switzerland) under incubation at 37 °C and with 5% CO_2_. The red (excitation: 535/9 nm, emission: 590/20 nm, number of flashes: 25)-to-green (excitation: 485/9 nm, emission: 535/20 nm, number of flashes: 25) ratio of JC-1 emissions was calculated for multiple reads per well (square, size: 3 × 3, border: 1200 µm, integration time: 20 µs).

### 2.7. Experimental Design

**Figure 1:**
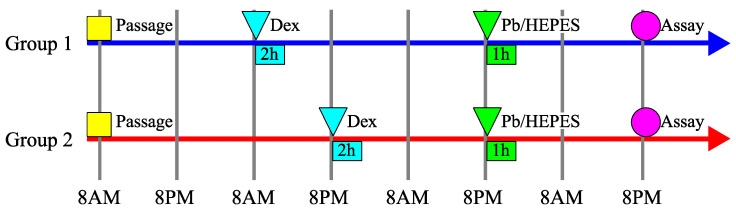
The timetable of the experimental design in the evaluation under lead (Pb) exposure. Dex: the reset of the circadian clock using dexamethasone shock for 2 h. Pb/HEPES: lead (Pb) exposure. Cells were seeded at 8 a.m., reset using dexamethasone shock the next day at 8 a.m. (Group 1, blue) or 8 p.m. (Group 2, red), treated with Pb the next day at 8 p.m. for 1 h, and then analyzed the day after that at 8 p.m.

Cells were seeded into microplates, and the circadian clock was reset using dexamethasone (Decadron phosphate; Sandoz Pharma K.K., Tokyo, Japan) shock for 2 h after 24 h (Group 1) and 36 h (Group 2). This was followed by overnight incubation. Then, 60 h after seeding, the cells were treated with HEPES buffer with or without Pb for 1 h and then incubated overnight again. The assays were performed 84 h after seeding. The time between Pb exposure and analysis was aligned to eliminate the influence of the recovery process after Pb-induced damage. Dexamethasone was previously reported to synchronize the circadian clock genes of olfactory tissues in mice [25]. Based on a previous study [10], 4 mM Pb was used. The timetable is shown in Figure 1. An amount of 0.9% saline solution (Otsuka Normal Saline; Otsuka Pharmaceutical, Tokyo, Japan) was used as the vehicle.

### 2.8. Statistical Analysis

All data are presented as mean ± standard deviation (SD) and were statistically evaluated in R version 4.4.2 [26]. Data were analyzed with Student’s *t*-test. A value of *p* < 0.05 was considered significant.

## 3. Results

### 3.1. Circadian Rhythm Was Reset After Dexamethasone Shock

**Figure 2:**
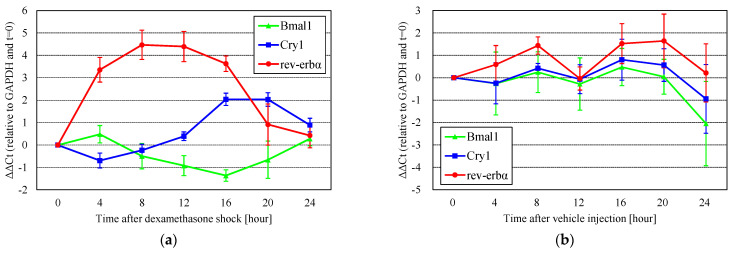
The rhythm of circadian clock gene expression after (**a**) dexamethasone shock (*n* = 4) or (**b**) vehicle injection (*n* = 3). Gene expression showed a daily rhythm after the reset using dexamethasone shock. Oscillations in gene expression after vehicle injection were also observed, but the differences in rev-erbα and Cry1 were smaller than with dexamethasone, and multiple peaks were observed.

Circadian clock genes showed a daily rhythm after the reset with dexamethasone shock (Figure 2a, using the Group 1 timetable of passage and dexamethasone shock). After the reset, the expression of rev-erbα or Cry1 was maximal after 8 h or 16 and 20 h, respectively. The change in Bmal1 expression was relatively small compared with that of rev-erbα and Cry1. These results indicate that dexamethasone shock is effective in resetting clock genes in DBC1.2 cells. The peak periods (8 h or 16 and 20 h) of rev-erbα or Cry1 expression and the bottom period (12–16 h) of Bmal1 expression were identical to those reported in previous studies [27,28]. Oscillations in gene expression after vehicle injection were also observed (Figure 2b), but the differences in rev-erbα and Cry1 were smaller than in dexamethasone shock, and multiple peaks were observed, indicating that clock gene oscillations are not stable, and their amplitudes are small without dexamethasone shock. Based on these results, we used dexamethasone to reset the clock genes.

**Figure 3:**
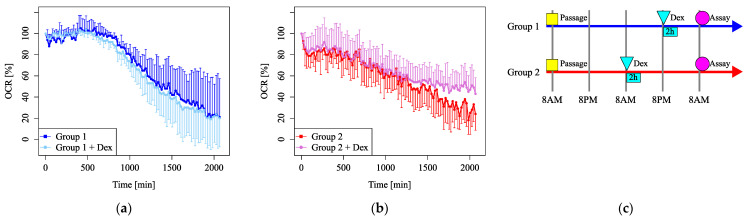
The long-term observation of the mitochondrial OCR after vehicle injection or dexamethasone shock. (**a**) The relative OCR in Group 1 with (light blue) or without (blue) dexamethasone shock displays values relative to time 0. The OCR gradually decreased due to the consumption of respiratory substrates in the medium. (**b**) The relative OCR in Group 2 with (orchid) or without (red) dexamethasone shock. The OCR showed diurnal variation and the OCR in Group 1 at about midday (720 min) was relatively high compared to the OCR in Group 2. The OCR at 1 day (1440 min) was comparable between the groups. Observations over 2 days were difficult due to the consumption of the medium and the inter-sample variation. (**c**) The timetable of the experimental design.

Vehicle injection did not markedly affect the clock gene cycle, but did affect mitochondrial OCR variation (Figure 3a,b). The OCR gradually decreased due to the consumption of respiratory substrates in the medium. The OCR showed diurnal variation within 1 day and the OCR in Group 1 at about midday (720 min) was relatively high compared to the OCR in Group 2, whereas the OCR at 1 day (1440 min) was comparable between the groups. Observations over 2 days were difficult due to the consumption of the medium and the inter-sample variation. These results suggest that medium exchange has a stronger effect on mitochondrial OCR changes than dexamethasone. In addition, the effect of medium replacement was estimated to become small in about one day.

### 3.2. Pb-Induced Mitochondrial Dysfunction

**Figure 4:**
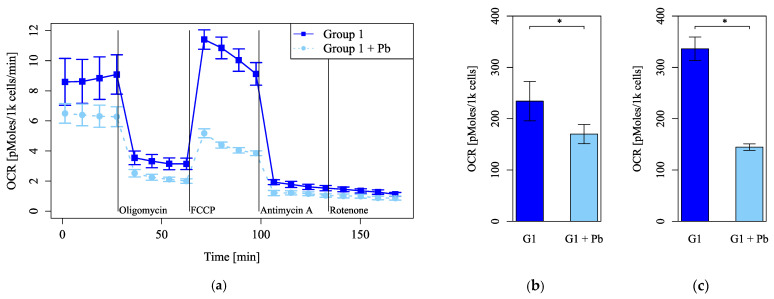
Mitochondrial dysfunction under lead (Pb) exposure. (**a**) Oxygen consumption rates (OCRs) were measured, followed by sequential treatment with oligomycin, FCCP, antimycin A, and rotenone. (**b**) The OCR of basal respiration. (**c**) The OCR of maximum respiration under FCCP injection (*n* = 5 per group, *: *p* < 0.05).

Mitochondrial function was evaluated by measuring the OCR and markedly decreased under Pb exposure (Figure 4a). Under FCCP injection, basal respiration (Figure 4b) and maximum respiration (Figure 4c) decreased significantly under Pb exposure. The maximum respiration under Pb exposure was less than half of that of the control (Figure 4c). These results indicate that Pb markedly injures mitochondrial function.

### 3.3. Pb-Induced Changes in Mitochondrial Function Depended on Circadian Rhythm

**Figure 5:**
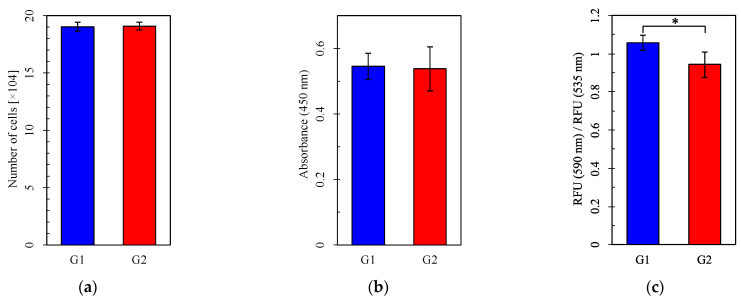

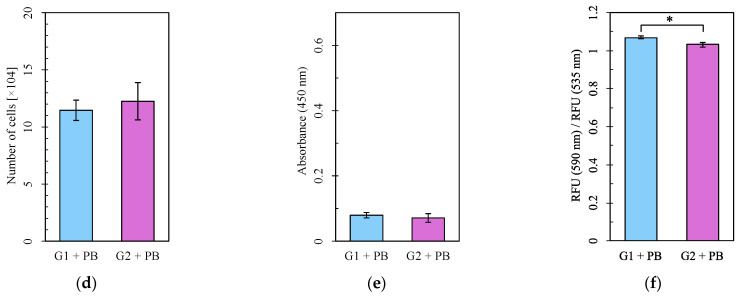
The number of cells, cell viability assay, and mitochondrial membrane potential assay. (**a**–**c**) Groups with no lead (Pb) exposure and (**d**–**f**) with Pb exposure. (**a**,**d**) Cell number. (**b**,**e**) Absorbance in the WST-8 assay. (**c**,**f**) RFU (590 nm)/RFU (535 nm) (emission). G1: Group 1, G2: Group 2, RFU: relative fluorescence units. For each group, *n* = 5 (**a**,**d**) or *n* = 6 (**b**,**c**,**e**,**f**). *: *p* < 0.05.

**Figure 6:**
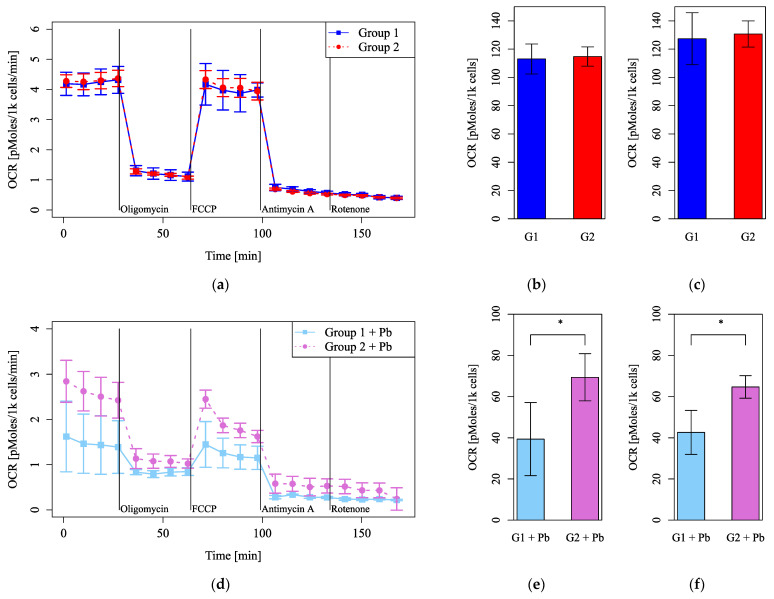
Mitochondrial function evaluated using the oxygen consumption rate (OCR) after dexamethasone shock. (**a**–**c**) Groups with no lead (Pb) exposure and (**d**–**f**) with Pb exposure. (**a**,**d**) The OCRs were measured, followed by sequential treatment with oligomycin, FCCP, antimycin A, and rotenone. (**b**,**e**) The OCR of basal respiration. (**c**,**f**) The OCR of maximum respiration under FCCP injection. G1: Group 1, G2: Group 2. *n* = 5 per group, *: *p* < 0.05.

Cell numbers and viability did not differ between Group 1 and Group 2 or between the Pb-treated and control groups (Figure 5a,b,d,e). However, MMP was significantly higher in Group 1 than Group 2, both without and with Pb exposure (Figure 5c,f). Mitochondrial function evaluated by measuring the OCR was more impaired under Pb exposure in Group 2 than in Group 1 (Figure 6d–f) but did not differ between Group 1 and Group 2 without Pb exposure (Figure 6a–c). Both basal respiration and the maximum OCR after FCCP injection were significantly lower in Group 2 than in Group 1 under Pb exposure (Figure 6e,f). These results suggest that Pb-induced mitochondrial damage was stronger in Group 1, whereas the circadian rhythm by itself did not affect mitochondrial respiration, as evaluated by measuring the OCR.

## 4. Discussion

In this study, we used dexamethasone to reset the synchronization of circadian clock genes in the DBC1.2 olfactory dark basal cell line, as was shown in other tissues and cell lines [29,30,31]. Mitochondrial function was significantly impaired in the Pb-treated group that was reset after 36 h compared with the Pb-treated group that was reset after 24 h. Cell numbers and viability did not differ between groups with altered circadian rhythms.

The timing of the reset using dexamethasone corresponds to the start of the activity cycle in both diurnal and nocturnal species [32]. Our results indicate that Pb-induced toxicity at the beginning of the activity period is more tolerable than at the beginning of the rest period. The cell line of a nocturnal species was used in this study, and the trigger of steroids indicates the onset of activity in peripheral organs. For a more comprehensive understanding, cell lines from diurnal species should also be investigated.

Circadian clock genes alter several downstream genes related to ER stress [33,34,35], mitochondrial functions [36,37,38], apoptosis [39,40], necrosis [41], and necroptosis [42]. The recovery of damaged cells depends on metabolic activity, which is affected by mitochondrial function [43]. The circadian rhythm regulates several transcriptional processes influencing cell metabolism, including mitochondrial activity, and the relationship between mitochondrial morphology and metabolic and energy rhythms is circadian-dependent [44]. The utilization of long-chain fatty acids by mitochondria depends on clock proteins, and the circadian clock regulates the diurnal utilization of various nutrients by mitochondria, optimizing mitochondrial responses to daily cycles of energy demands [45]. The necroptosis pathway in Pb-induced cytotoxicity is altered by circadian clock genes [10], whereas mitochondrial metabolism is modulated by many pathways, which could be the subject of future studies.

Circadian functional modulation is also reported at the organ level. The circadian clock system plays an important role in regulating mitochondrial metabolism and thereby maintains cardiac function [46]. Circadian-rhythm-dependent changes in sensitivity to various chemical agents have been reported, and this diurnal modulation is also applied in clinical settings and is called chronotherapy [47]. Optimizing the timing of chemotherapy can reduce drug toxicity and increase efficacy. More attention could be placed on the protective treatment of olfactory organs during the highly sensitive period, which may be at the end of the activity period, based on the results of this study.

In general, a high MMP has been reported to indicate good mitochondrial status, which implicates a high OCR [48]. However, the results of this study showed the opposite, with a high MMP in Group 1 with Pb and a high OCR in Group 2 with Pb. In a previous study evaluating adaptations in energy metabolism in rat cortical neurons, potassium affected both the OCR and MMP, whereas glutamate affected only the OCR [49], and increasing the Ca^2+^ concentration by inhibiting ATPase of the ER using thapsigargin affected only MMP. These results of this study indicated that different metabolic pathways have very different effects on MMP fluctuation. The proton leak may be involved in metabolic adaptations [50]. In a myocardial postischemic model, an increasing OCR with a decreasing MMP was observed after reperfusion [51]. In practice, MMP is difficult to assess, and an increase in the OCR relates to a slight decrease in MMP under specific conditions [52]. Thus, mainly OCR data were interpreted in this study.

MMP exhibits circadian patterns and is associated with mitochondrial activity [53]. MMP is higher during the light period (specifically the end of the light period) than the dark period in the suprachiasmatic nucleus (SCN) of rats [54]. In both diurnal and nocturnal species, the peak of metabolic activity in the SCN is during the day, and peripheral organs show peaks either during the day (in diurnal species) or during the night (in nocturnal species) [36,37,38]. In this study, MMP was higher in Group 1 than in Group 2, and the assay of Group 1 was at the point of transition from the active to the terminal phase. This finding is consistent with the previous study [54] but requires additional time points for precise validation.

## 5. Conclusions

The circadian rhythm can alter sensitivity to Pb-induced toxicity in olfactory cells and can modify mitochondrial damage. The importance of daily cycles in olfactory cell recovery suggests the possibility of optimal treatment timings in clinical settings.

## Data Availability

The raw data supporting the conclusions of this article will be made available by the authors on request.

## Appendix A

The ImageJ macro for counting cells.

function fillZero(num, lim) {

  if (lengthOf(num) < lim) { out = "";

    for (_i = 0;_i < lim-lengthOf(num);_i ++) { out += "0"; }

out += num; return out; } else return num; }

function countNuclei(filenamepf, startnum, endnum) {

 for (i = startnum;i <= endnum;i ++) {

  open(filenamepf + fillZero(""+i,2) + ".tif");

  ititle = getTitle();

  selectWindow(ititle);

  run("Smooth"); run("Smooth"); run("8-bit");

  run("Auto Local Threshold",

    "method=Bernsen radius=10 parameter_1=0 parameter_2=0 white");

  run("Options...", "iterations=1 count=1 black do=Nothing");

  run("Watershed");

  run("Analyze Particles...", "exclude include summarize");

  stitle = getTitle();

  saveAs("Results", filenamepf + fillZero(""+i,2) + ".txt");

  selectWindow("" + fillZero(""+i,2) + ".txt"); run("Close");

  selectWindow(ititle); saveAs("Tiff", filenamepf + fillZero(""+i,2) + "_proc.tif"); close();

 }

}

countNuclei("C:/Users/USER1/Desktop/tk/20240905_XF24/20230925/", 1, 5);

## Appendix B

GAPDH

forward: 5′-CATGGCCTTCCGTGTTCCTA-3′

reverse: 5′-CCTGCTTCACCACCTTCTTGA-3′

probe 5′-HEX-CCGCCTGGAGAAACCTGCCAAGTATG-BHQ-1-3′

Bmal1

forward: 5′-CCAAGAAAGTATGGACACAGACAAA-3′

reverse: 5′-GCATTCTTGATCCTTCCTTGGT-3′

probe: 5′-6-FAM-TGACCCTCATGGAAGGTTAGAATATGCAGAA-BHQ1-3′

Cry1

forward: 5′-CTGGCGTGGAAGTCATCGT-3′

reverse: 5′ -CTGTCCGCCATTGAGTTCTATG-3′

probe: 5′-TAMRA-CGCATTTCACATACACTGTATGACCTGGACA-BHQ2-3′

Rev-erbα

forward: 5′-CATGGTGCTACTGTGTAAGGTGTGT-3′

reverse: 5′-CACAGGCGTGCACTCCATAG-3′

probe: 5′-Cyanine5-ACGTGGCCTCAGGC-BHQ2-3′
